# Noise and the Perceptual Filling-in effect

**DOI:** 10.1038/srep24938

**Published:** 2016-04-22

**Authors:** Ativ Zomet, Uri Polat, Dennis M. Levi

**Affiliations:** 1 Goldschleger Eye Research Institute, Faculty of Medicine, Tel-Aviv University, Israel; 2 School of Optometry, UC Berkeley, Berkeley, CA, USA; 3 School of Optometry & Visual Science, Bar Ilan University, Israel

## Abstract

Nearby collinear flankers increase the false alarm rate (reports of the target being present when it is not) in a Yes-No experiment. This effect has been attributed to “filling-in” of the target location due to increased activity induced by the flankers. According to signal detection theory, false alarms are attributed to noise in the visual nervous system. Here we investigated the effect of external noise on the filling-in effect by adding white noise to a low contrast Gabor target presented between two collinear Gabor flankers at a range of target-flanker separations. External noise modulates the filling-in effect, reducing visual sensitivity (d′) and increasing the filling-in effect (False Alarm rate). We estimated the amount of external noise at which the false alarm rate increases by the √2 (which we refer to as N_FA_). Across flank distances, both the false alarm rate and d′ (with no external noise) are correlated with N_FA_. These results are consistent with the notion that nearby collinear flankers add both signal and noise to the target location. The increased signal results in higher d′ values; the increased noise to higher false alarm rates (the filling effect).

Visual sensitivity is limited by both the strength of the neural signals, and the noise in the visual nervous system^1^. Measuring visual performance in noise can provide useful insights into the neural mechanisms and computations used to solve a visual task^2^,^3^,^4^. For example, in the “classical” noise paradigm^5^,^6^,^7^, external noise is added to the display screen, while the observer performs a visual task. The equivalent input noise is the level of external noise that raises the threshold by a criterion amount (e.g. √2) relative to the no noise condition. This approach has been widely used to characterize the limitations imposed by internal noise in the visual system^3^,^7^,^8^,^9^,^10^,^11^,^12^,^13^,^14^.

When observers are asked to report the presence (yes) or absence (no) of a near threshold target (a Gabor patch) both their hit rate (reporting “yes” on target present trials) and their false alarm rate (reporting “yes” on target absent trials) increase in the presence of nearby collinear flankers^15^,^16^,^17^,^18^,^19^. The increased sensitivity induced by nearby flankers has been explored in many previous studies^15^,^20^,^21^,^22^,^23^,^24^,^25^,^26^,^27^. However, the flanker-induced hallucinations (increased false alarm rate) have been less explored. Zomet *et al*. refer to the flanker-induced hallucinations as the “filling-in effect”. Interestingly, patients with major depression exhibit an impaired filling-in effect^16^. One interpretation for the filling-in effect is that collinear flankers produce neuronal activity, via lateral interactions, at locations corresponding to the target even if it is not directly activated by feed forward input^15^,^19^,^28^. An alternative view is that the high contrast flankers directly stimulate neurons that are involved in detecting the target (due to overlapping receptive fields and fixational eye movements) and thus act as a low contrast pedestal^29^.

In this study we explored how noise affects the filling-in effect. We varied the amount of external noise (containing a broad range of random orientations and spatial frequencies) while measuring the filling-in effect, similar to previous studies^15^,^16^,^17^,^18^,^20^,^21^. Specifically, we estimated the external noise level at which the false alarm rate increases by a criterion amount −√2 – we term this N_FA_ - for detection of a Gabor target presented between two collinear Gabor flankers. To anticipate, we found that across flank distances, both the false alarm rate and d′ (with no external noise) are correlated with N_FA_.

## Results

**Figure 1:**
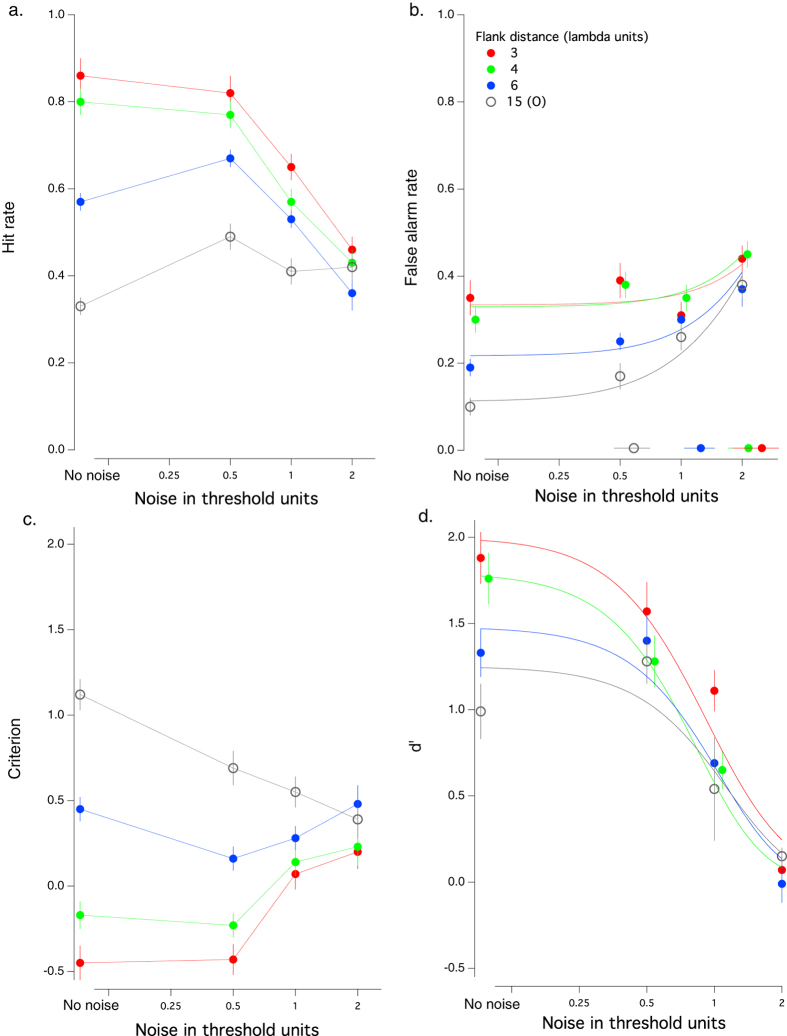
The effect of adding external noise on hit rate (**a**) false alarm rate (**b**) criterion (**c**) and d′ (**d**). Mean data of 16 observers for 4 flank distances. The curves fit to the false alarm data (**b**) are equivalent input noise curves (see text), and the symbols along the abscissa represent N_FA_ for each flank distance (see text for details).

Figure 1 shows the effect of noise on the mean hit rate (a) false alarm rate (b) criterion (c) and d′ (d) for each target-to-flank separation. With low noise (the two leftmost points in each panel), the data replicates the previous studies of this filling-in^15^,^16^,^20^,^21^ - hit rate, false alarm rate, and d′ increase systematically as the flank distance decreases, and the criterion decreases (becomes less positive).

Importantly, the effect of added noise depends on both the noise level and the flank distance. With distant, orthogonal flankers (15λ open gray symbols), adding noise results in a significantly increased False Alarm rate (F(3,60) = 6.503, P = 0.001), reduced d′ (F(3,60) = 5.445, P=0.002), reduced criterion (F(3,60) = 3.319, P = 0.026) but no significant change in hit rate (F(3,60) = 1.395, P = 0.253). In contrast, with close flankers, adding noise significantly reduces the hit rate (3λ – red circles, F(3,60) = 15.044, P = 0.000; 4λ – green circles, F(3,60) = 12.867, P = 0.000) and d′ (3λ; F(3,60) = 11.08, P = 0.000, 4λ; F(3,60) = 12.129, P = 0.000), with little or no effect on False Alarms (3λ, F(3,60) = 0.867, P = 0.463, 4λ; F(3,60) = 1.511, P = 0.221).

Once visible (the two rightmost points in each panel), the external noise reduces the hit rate (except for the orthogonal flanks), increases the false alarm rate and reduces d′. Increasing the noise also alters the criterion, making it more positive for the smaller flank separations, and more negative for the large orthogonal.

**Figure 2:**
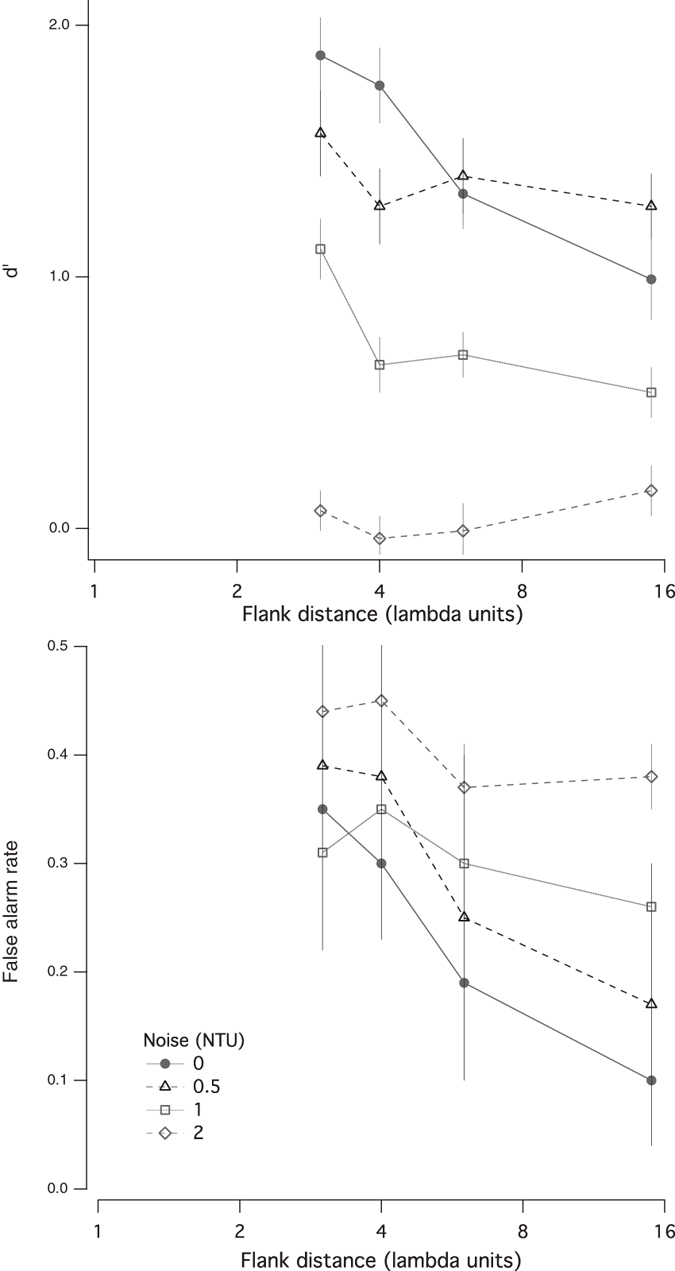
The effects of flank distance on d′ (top) and false alarm rate (bottom) for 4 different noise levels.

The effects of flank distance on d′ and false alarm rate for different noise levels can be seen more easily in Fig. 2. For low levels of noise (filled circles and open squares), the results are consistent with previously reported effects of facilitation as a function of target-flanker separations^15^,^16^,^17^,^19^,^20^,^21^. At twice the noise detection threshold (open diamonds), facilitation is abolished (d′ is independent of flank distance), and the false alarm rate is high at all flank distances. We note that while the absence of facilitation at small flanker separations and high noise levels may be attributed to a ‘floor’ effect (d′ for the other conditions cannot go below zero), facilitation is also absent in contrast discrimination experiments when the pedestal is well above threshold^30^,^31^.

## Discussion

Our results show that adding external noise to the stimulus modulates the filling-in effect. In short, increasing external noise reduces visual sensitivity (d′) and increases the false alarm rate. Increasing flank distance (in the absence of external noise) reduces visual sensitivity (d′) and decreases the false alarm rate. The effect of external noise on sensitivity is well understood, and the increased false alarm rate is not surprising, since samples of the broadband noise can mimic the signal. Indeed, this is the basis of the image classification technique^1^,^4^. The effect of flankers has been well documented, but is less well understood. Specifically, in the absence of external noise, nearby flankers facilitate detection (Fig. 1d, left most data) and increase the false alarm rate (Fig. 1b, left most data). The current results document the interaction between flank distance and the false alarm rate.

### How much external noise is required to change the false alarm rate?

It is clear from Fig. 1b that at large flank distances (open circles), with no noise the false alarm rate is low, and begins to increase at relatively low noise levels, whereas at small flank distances (red and green circles), the false alarm rate is high, and only rises when the noise is highly visible. To quantify the effect of noise on the false alarm rate we fit the data in Fig. 1b (False Alarm rate vs. Noise) with a function of the form: FA = k √(σ_E_^2^ + N_FA_^2^), where k is a multiplicative constant; σ_e_ is the external noise and N_FA_ is the external noise level at which the false alarm rate increases by a criterion amount −√2 (the smooth curves in Fig. 1b). The symbols along the abscissa show N_FA_ for each flank distance. The astute reader may notice a close resemblance between this formula and the classical equivalent noise function^5^; however, we do not mean to apply the equivalent noise framework. Rather, it provides a reasonable fit (as determined by chi square) to the data and we use it here simply to determine the strength of external noise that raises the false alarm rate by √2.

**Figure 3:**
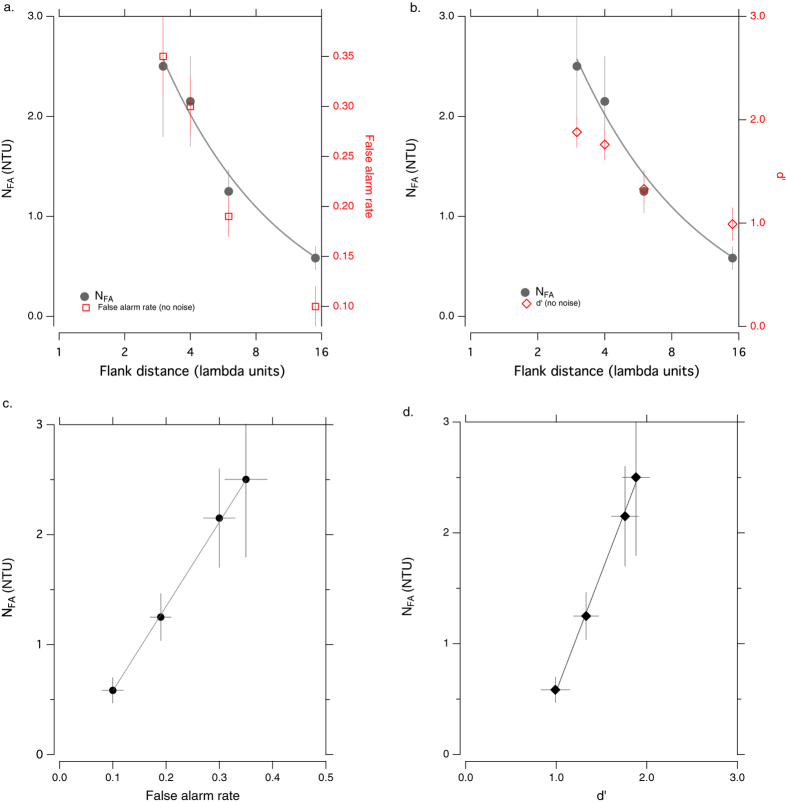
The effect of flank distance on: (**a**) N_FA_ (left ordinate) and false alarm rate (right ordinate). (**b**) N_FA_ (left ordinate) and d′ (right ordinate). (**c**) The relationship between N_FA_ and false alarm rate, and (**d**) The relationship between N_FA_ and d′.

Figure 3a,b (gray circles and left ordinate) show that N_FA_ increases systematically as flank distance is reduced. The red symbols and right ordinate show similar increases in false alarm rate (3a) and d′ (3b) with no added external noise. Importantly, N_FA_ is significantly correlated with both false alarm rate and d′ (Fig. 3c,d respectively −r = 0.82 ± 0.03 (p < 0.001) for N_FA_ vs FA ; r = 0.38 ± 0.02 (p = 0.004) for N_FA_ vs d′). A strong correlation between N_FA_ and false alarm rate is to be expected (since it is derived from FA), and the relationship between False alarm rates and d′ is well known. Nevertheless, the correlation between N_FA_ (the external noise level at which the false alarm rate increases by the √2) and d′ at different flank distances, is somewhat surprising.

These results are consistent with the notion that nearby collinear flankers add both signal and noise to the target location. The increased signal results in higher d′ values; the increased noise to higher false alarm rates (the filling-in effect), and elevated N_FA_, similar to the effect of adding external noise to the stimulus, which mimics the intrinsic noise in the visual system.

### Relation to Major Depression Disorder (MDD)

In previous study we found that patients with major depression exhibit an impaired filling-in effect^16^, and reduced suppression in a contrast discrimination task^32^. Specifically, the patients exhibited lower hit and false alarm rates than normal control subjects. These results were explained in terms of deficits in neural excitation and increased levels of inhibition that may prevent the neurons from reaching saturation^33^. An alternative suggestion was that MDD patients may have high level of internal noise. However, we did not find any filling-in effect at larger target-flanker separations, as was found here at high levels of external noise. Thus, it seems that the noise explanation is unlikely to account for the impaired filling-in effect in patients with depression rather, it may support the idea that the filling-in effect results from neural excitation in the early visual areas of the visual system.

## Methods and Materials

### Participants and procedures

Sixteen adults, between the ages of 21 and 38 (mean age 26.06 ± 4.74), with normal or corrected-to-normal visual acuity (6/6) participated in these experiments. All participants gave written informed consent to prticipate in the study, and the experiment performed under the relevant laws and institutional guidelines of UC Berkeley IRB committee (CPHS). All experimental protocol were performed in accordance with the guidelines provided by the committee approving the experiments.

### Stimuli and Methods

**Figure 4:**
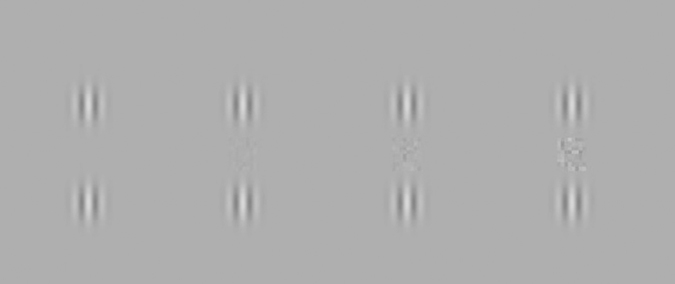
The stimuli used in this study were collinear configurations with different target flanker separations (3, 4, 6, and 15 lambda, λ), and external white noise; the target-flanker separation in this figure is 3λ, and the noise level is 0, 0.5, 1, and 2 (left to right) of the noise threshold. The target absolute threshold in this figure is 3, and the noise absolute threshold is 4.

The method was similar to the paradigm reported previously (without noise^15^,^16^,^18^). The task required participants to detect a low contrast Gabor target that was presented between two high contrast (c = 60%) lateral collinear flankers (Gabor patches, see Fig. 4). The target was randomly presented in 50% of the trials. The target was flanked above and below by high contrast collinear flankers with the same spatial frequency (6 cycles per degree) and orientation (vertical) at different target-flanker separations of 3, 4, 6, lambda, (λ), and with orthogonal flankers (not collinear) at a target-flanker separation of 15λ, in order to estimate the detection of the target under a condition of no facilitation. The contrast of the target (Gabor patch) was set for each participant to his/her detection threshold (with no noise), estimated using an adaptive staircase method (79% correct), with orthogonal flankers (not collinear) at a target-flanker separation of 15λ. Stimuli were displayed on a Sony multiscan G400 color monitor (1024 × 768 pixels at a 75 Hz refresh rate; gamma correction applied) controlled by a PC with a mean luminance of 40 cd/m^2^. The effective size of the monitor was 26 × 35 cm, which at a viewing distance of 150 cm subtended a visual angle of 9.7 × 11.4 degrees.

White noise (containing a broad range of random orientations and spatial frequencies) at differing levels of contrast was presented at the target location and was superimposed on the target when present. For each participant, the noise contrast was normalized to his/her noise threshold detection threshold, measured separately estimated using an adaptive staircase method (79% correct), and was presented at 0, 0.5, 1, and 2 times their noise detection threshold. In the Results section we quantify the noise strength in noise threshold units (NTU).

Participants had to report whether the target was present (Yes) or absent (No) by pressing the left and right mouse keys, respectively. For a given noise level, the stimuli were presented in random order and all target-flanker separations were mixed (mixed-by-trials). Each block consisted of 20 trials at each of the four target-flanker separations (80 trials per block). There were four blocks, each with a different external noise level (0, 0.5, 1, and 2 NTU). The starting noise level was randomized between participants.

Participants repeated each noise level three times for a total of 960 trials (20 × 4 flank distances X 4 noise levels X 3 repetitions).

To reduce position uncertainty, a white fixation circle on grey background (size 0.23 degrees) indicated the location of the target, and the participants activated the sequence of presentation of the trials at their own pace. The briefly displayed stimuli (100 msec.) were viewed binocularly, and auditory error feedback was provided after each presentation.

The false alarm (FA), Miss, Hit, and correct rejection were recorded and analyzed, yielding the sensitivity (d′ = z(Hit) - z(FA)) and the criterion (Cr = −0.5[z(Hit) + z(FA)] measures, with z defined as the inverse of the normal distribution function. This calculation was used in previous studies^15^,^16^,^17^,^18^ and is based on MacMillan and Creelman’s equation^34^, which can be viewed as a deviation from the ideal observer’s decision criterion. Our detection model is based on a single criterion, but note that we are not able to distinguish between this model and a multiple criterion model.

The correlations presented in the Discussion are based on fitting the data of each observer individually with the equivalent noise model, and calculating the correlation between NFA and FA and NFA and d′ separately for each individual. The values reported represent the median of those correlations.

## Additional Information

**How to cite this article**: Zomet, A. *et al*. Noise and the Perceptual Filling-in effect. *Sci. Rep.* **6**, 24938; doi: 10.1038/srep24938 (2016).
